# Flat Magnetic Stimulation in the Conservative Management of Mild Pelvic Organ Prolapse: A Retrospective Observational Study

**DOI:** 10.3390/medicina61122198

**Published:** 2025-12-11

**Authors:** Desirèe De Vicari, Marta Barba, Alice Cola, Nicola Amatucci, Sebastiano Carrara, Matteo Frigerio

**Affiliations:** Department of Gynecology, IRCCS San Gerardo dei Tintori, University of Milano-Bicocca, 20900 Monza, Italy; d.devicari@campus.unimib.it (D.D.V.); m.barba8792@gmail.com (M.B.); alice.cola1@gmail.com (A.C.); n.amatucci@campus.unimib.it (N.A.)

**Keywords:** pelvic organ prolapse, flat magnetic stimulation, conservative management, pelvic floor rehabilitation, non-invasive therapy

## Abstract

*Background and Objectives:* Pelvic organ prolapse (POP) is a prevalent pelvic floor disorder affecting a large proportion of parous and aging women worldwide. While surgical intervention is generally reserved for advanced prolapse, conservative approaches remain essential for the management of early-stage disease. Flat magnetic stimulation (FMS), a novel non-invasive modality, has shown promising results in pelvic floor rehabilitation for urinary incontinence, but its role in prolapse treatment remains insufficiently investigated. This study aimed to evaluate anatomical and patient-reported outcomes in women with mild POP undergoing FMS therapy. *Materials and Methods:* This retrospective observational study included 87 women with stage ≤ 2 POP, classified according to the Pelvic Organ Prolapse Quantification (POP-Q) system. Participants underwent eight FMS sessions, each lasting 25 min, over four weeks using the Dr. Arnold device (DEKA, Calenzano, Italy). Pre- and post-treatment evaluations included standardized POP-Q measurements and the Patient Global Impression of Improvement (PGI-I) questionnaire. Statistical analyses were performed using paired *t*-tests, with significance set at *p* < 0.05. *Results:* Statistically significant improvements were observed in the anterior vaginal compartment, with mean Aa values improving from −0.3 ± 1.2 to −0.7 ± 1.3 (mean difference −0.4 cm; 95% CI −0.8 to −0.03; *p* = 0.03; Cohen’s d = 0.31) and mean Ba values from −0.3 ± 1.3 to −0.7 ± 1.3 (mean difference −0.4 cm; 95% CI −0.8 to −0.02; *p* = 0.04; Cohen’s d = 0.30). No significant changes were found at other POP-Q landmarks. According to PGI-I results, 90.8% of participants reported symptom improvements, and 37.9% described their condition as “very much improved” or “much improved.” No adverse events occurred, and treatment compliance was 100%. *Conclusions:* FMS seems to be a safe, well-tolerated, and potentially effective conservative therapy strategy for mild POP, offering both objective anatomical benefits and high subjective satisfaction. Further randomized controlled trials with longer follow-up are required to validate these findings and clarify the long-term role of FMS in the management of pelvic floor dysfunction.

## 1. Introduction

Pelvic organ prolapse (POP) constitutes a frequent manifestation of pelvic floor dysfunction, resulting from the attenuation or failure of connective and muscular support for pelvic viscera [1]. This leads to descent or protrusion of organs such as the bladder, uterus, or rectum into or beyond the vaginal canal. POP has been reported to affect up to 50% of parous women during their lifetime, with incidence rates increasing substantially in the postmenopausal demographic [1,2,3].

Risk factors for POP are multifactorial and include obstetric history, advancing age, obesity, chronic increases in intra-abdominal pressure, and inherent connective tissue disorders [4,5]. The condition’s symptomatology varies but typically includes pelvic pressure, vaginal bulging, urinary and fecal incontinence, incomplete voiding, and sexual dysfunction, all of which can profoundly impair quality of life (QoL) [6,7].

Management strategies for POP are highly individualized. While surgical interventions offer definitive correction for advanced prolapse, conservative options are emphasized in early stages to manage symptoms, preserve function, and delay or avoid surgery [8,9,10,11]. Pelvic floor muscle training (PFMT) is widely endorsed as a first-line conservative treatment, yet adherence remains suboptimal due to time constraints, discomfort, or lack of perceived benefit [8,9,10].

Another widely used conservative approach is the application of vaginal pessaries, including ring and occlusion types. These devices provide mechanical support to the prolapsed organs, alleviating symptoms such as vaginal bulging, pelvic pressure, and urinary difficulties. Pessaries can be fitted and managed by trained clinicians or physiotherapists, offering an effective non-surgical option for women who are either unsuitable for surgery or prefer to delay operative intervention. The use of pessaries can be combined with PFMT to optimize pelvic floor support and improve patient-reported outcomes [2,3,8].

In this context, magnetic stimulation (MS) offers a non-invasive therapeutic alternative. MS utilizes time-varying electromagnetic fields to elicit deep pelvic muscle contractions without requiring internal probes, increasing patient comfort and compliance [12,13,14,15]. Flat Magnetic Stimulation (FMS), a more recent technological advancement, delivers a uniform electromagnetic field over a broader area of the pelvic floor, potentially enhancing efficacy and coverage of key support muscles such as the pubococcygeus and iliococcygeus [16,17,18,19,20].

Though extensively studied in the context of urinary incontinence [16,17,21,22,23], the role of FMS in prolapse management remains understudied. This study addresses this gap by evaluating both objective anatomical improvements and subjective patient-reported outcomes following FMS therapy in women with mild POP. Although surgery represents the definitive treatment for advanced stages of prolapse, conservative approaches remain pivotal in early stages, particularly physiotherapy-based interventions. Pelvic floor muscle training (PFMT), delivered under the supervision of specialized physiotherapists, is considered the cornerstone of conservative management, with robust evidence from randomized controlled trials and meta-analyses demonstrating improvement in symptoms and quality of life [8,9,10,11]. However, adherence can be challenging, and outcomes are highly dependent on patient engagement and training quality [9,10]. In recent years, magnetic stimulation (MS) has emerged as a non-invasive physiotherapeutic option, inducing pelvic floor contractions without the discomfort of intravaginal probes, thereby enhancing patient comfort and compliance [13,14,15]. Flat Magnetic Stimulation (FMS), an innovative evolution of MS, delivers a uniform electromagnetic field across the pelvic floor, producing supramaximal muscle contractions and potentially offering broader and deeper activation of the pubococcygeus, iliococcygeus, and associated structures [18,19,20].

Clinical studies have consistently shown the effectiveness of FMS in the treatment of urinary incontinence (UI), including urge, stress, and mixed types, with improvements in both objective measures and patient-reported outcomes [16,17,19,20,23]. These studies highlight the safety, tolerability, and therapeutic benefits of FMS in pelvic floor dysfunctions. However, despite this growing body of evidence in UI, its role in pelvic organ prolapse (POP) remains largely unexplored. To date, no study has systematically investigated the impact of FMS on anatomical outcomes in prolapse, representing a significant gap in the literature. The present study addresses this gap by evaluating both objective anatomical changes and subjective improvements in women with mild POP following FMS therapy.

## 2. Materials and Methods

### 2.1. Study Design and Participants

This retrospective observational study was conducted between January 2022 and December 2023 at the Gynecology Department of San Gerardo Hospital, Monza, Italy. Women aged 30 to 75 years presenting with symptomatic pelvic organ prolapse (POP) classified as stage ≤ 2 according to the Pelvic Organ Prolapse Quantification (POP-Q) system [21] were eligible for inclusion. Exclusion criteria comprised previous pelvic reconstructive surgery for POP, neurological disorders, pelvic malignancies, presence of a cardiac pacemaker, or history of pelvic irradiation. Written informed consent was obtained from all participants prior to enrollment. The study protocol was reviewed and approved by the local Institutional Review Board (ASST Monza; protocol code MAGCHAIR) and was conducted in accordance with the Declaration of Helsinki.

### 2.2. POP-Q Assessment

The POP-Q system, which represents the international gold standard for the evaluation and staging of pelvic organ prolapse, was employed for anatomical assessment [21]. The system quantifies descent at specific vaginal reference points (Aa, Ba, C, Ap, Bp, and D) in centimeters relative to the hymenal plane. Examinations were carried out by a single experienced urogynecologist at baseline (T0) and at follow-up (T1), performed one week after completion of the intervention, in order to minimize inter-observer variability and ensure consistency in measurements.

### 2.3. Intervention: Flat Magnetic Stimulation

The therapeutic protocol consisted of eight sessions of Flat Magnetic Stimulation (FMS) delivered with the Dr. Arnold chair device (DEKA, Calenzano, Italy). Each session lasted 25 min and was administered twice weekly over a period of four consecutive weeks. Participants remained fully clothed and seated during treatment, and neither anesthesia nor invasive instrumentation was required.

The device generates a homogeneous electromagnetic field distributed uniformly across the pelvic floor. This induces supramaximal contractions of the levator ani muscle complex and associated connective tissue structures, thereby promoting neuromuscular re-education, enhancing muscular tone, and improving local vascularization. The underlying physiological rationale derives from previous studies demonstrating the efficacy of FMS in urinary incontinence (UI), where significant improvements in pelvic floor muscle function and symptomatology were reported [16,17,22,23].

### 2.4. Outcome Measures

The primary outcome measure was the change in anatomical support as quantified by POP-Q point values (Aa, Ba, C, Ap, Bp, D) between baseline and follow-up. The secondary outcome was subjective improvement in symptoms, assessed using the Patient Global Impression of Improvement (PGI-I) questionnaire, a validated seven-point Likert scale ranging from 1 (“very much improved”) to 7 (“very much worse”), where scores of 1–3 were considered to represent clinically meaningful improvement [24].

### 2.5. Consent and Questionnaire Administration

Written informed consent was obtained from all participants prior to enrollment. The Patient Global Impression of Improvement (PGI-I) questionnaire, a validated seven-point Likert scale [24], was administered one week after the completion of the treatment cycle. The administration time was approximately 5–7 min per patient, and the questionnaire was used in the validated Italian version for language and completeness.

### 2.6. Statistical Analysis

Continuous variables were expressed as mean ± standard deviation (SD). Comparisons of POP-Q measurements before and after treatment were performed using paired *t*-tests. A *p*-value < 0.05 was considered indicative of statistical significance. Patient-reported outcomes measured through PGI-I scores were analyzed descriptively to provide a summary of subjective response to treatment.

## 3. Results

A total of 87 women met the inclusion criteria and completed the entire Flat Magnetic Stimulation (FMS) treatment protocol without any dropouts or missing data. The mean age of the cohort was 59.1 years (±6.4), with all participants attending the full course of eight sessions over four consecutive weeks. Treatment adherence was excellent, and no patient reported difficulty in tolerating the therapy. Moreover, no adverse events, discomfort, or complications were observed at any stage during or after the sessions, highlighting the high safety profile, feasibility, and overall acceptability of FMS as a non-invasive intervention for women with mild pelvic organ prolapse (POP) (Table 1).

### 3.1. Anatomical Outcomes

Objective anatomical improvements were evaluated by comparing pre- and post-treatment POP-Q measurements, with particular attention to the anterior vaginal compartment, where prolapse was most commonly localized among study participants. Statistically significant improvements were observed at points Aa and Ba, indicating a measurable elevation of the anterior vaginal wall. Specifically, the Aa point improved from a baseline mean of −0.3 ± 1.2 to −0.7 ± 1.3 (mean difference −0.4 cm; 95% CI −0.8 to −0.03; *p* = 0.03; Cohen’s d = 0.31), while the Ba point shifted from −0.3 ± 1.3 to −0.7 ± 1.3 (mean difference −0.4 cm; 95% CI −0.8 to −0.02; *p* = 0.04; Cohen’s d = 0.30). These changes suggest that FMS may enhance anterior pelvic support, possibly through induced neuromuscular activation and strengthening of pelvic floor structures such as the pubococcygeus and iliococcygeus muscles.

No statistically significant changes were observed in the apical compartment (point C: −5.4 ± 2.5 to −5.5 ± 2.4; *p* = 0.59; and point D: −7.0 ± 2.3 to −7.0 ± 2.3; *p* = 0.94), nor in the posterior vaginal wall (Ap: −1.1 ± 1.3 to −1.2 ± 1.4; *p* = 0.42, and Bp: −1.2 ± 1.4 to −1.3 ± 1.5; *p* = 0.38) (Table 2). These findings suggest that the therapeutic effects of FMS, at least in the short term, may be more pronounced in the anterior compartment, possibly reflecting the dominant localization of mild prolapse in this cohort or the anatomical distribution of muscle engagement during FMS. These results also support the hypothesis that the device’s electromagnetic field is most effective in stimulating structures adjacent to the pubic symphysis, with limited influence on deeper apical or posterior elements.

### 3.2. Subjective Outcomes (PGI-I Scores)

**Figure 1:**
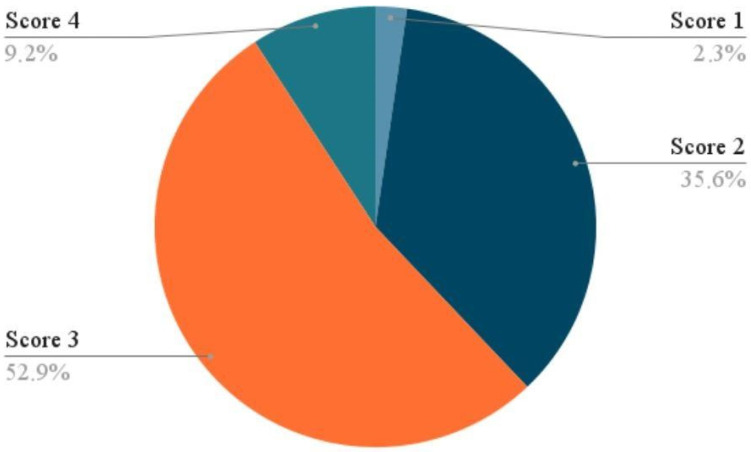
Patient Global Impression of Improvement (PGI-I) Scores.

In addition to anatomical changes, subjective symptom relief was assessed using the Patient Global Impression of Improvement (PGI-I) scale, administered one week after the completion of the FMS treatment cycle. At follow-up (T1), 90.8% of patients (*n* = 79) reported a degree of symptomatic improvement, defined as a PGI-I score between 1 and 3. Among these, 2.29% (*n* = 2) rated themselves as “very much improved” (score 1), while 35.63% (*n* = 31) selected “much improved” (score 2), and 52.87% (*n* = 46) reported being “minimally improved” (score 3). Only a small subset of participants, 9.2% (*n* = 8), reported no perceptible change in symptoms (score 4), and notably, none of the participants reported worsening of their condition (scores 5 to 7), indicating both therapeutic benefit and a lack of negative outcomes. (Figure 1)

These subjective outcomes, combined with the favorable anatomical improvements and absence of adverse events, underscore the safety and acceptability of FMS in this patient population. The treatment’s non-invasive nature, involving no internal manipulation or anesthesia, likely contributed to the high satisfaction and adherence observed. Additionally, the uniformity of electromagnetic field delivery and comfort of the seated treatment position may have enhanced overall patient experience, making FMS a promising adjunct or alternative to traditional conservative therapies such as pelvic floor muscle training.

## 4. Discussion

The findings from this retrospective observational study suggest that Flat Magnetic Stimulation (FMS) represents a promising, non-invasive therapeutic modality for the conservative management of mild pelvic organ prolapse (POP). Statistically significant improvements were observed in the anterior vaginal wall—specifically at Pelvic Organ Prolapse Quantification (POP-Q) points Aa and Ba—demonstrating a measurable enhancement in pelvic support, consistent with the hypothesized mechanism of action of FMS. By generating homogeneous electromagnetic fields, the FMS device is designed to elicit supramaximal contractions of pelvic floor muscles, potentially engaging key muscular structures such as the pubococcygeus, iliococcygeus, and associated connective tissue networks. These contractions are likely to contribute to enhanced muscular tone, increased local vascular perfusion, and neuromuscular re-education, all of which constitute foundational components in the conservative management of pelvic floor disorders.

The results of this study underscore the potential of FMS as a conservative treatment option for mild POP. Objective improvements in the anterior vaginal wall at POP-Q points Aa and Ba suggest a targeted anatomical response consistent with the underlying mechanism of electromagnetic-induced muscle contractions. The anterior compartment is frequently the earliest and most commonly affected site in early stage prolapse; therefore, the observed anatomical benefits are both clinically and mechanistically plausible [25,26,27,28]. The absence of significant changes in posterior or apical compartments further supports the specificity of the intervention. Subjectively, a high proportion of patients reported improvement—particularly substantial improvement—indicating strong patient-perceived benefit. The absence of adverse events and complete adherence among all patients reinforce the safety and acceptability of this modality. Compared with traditional magnetic stimulation, the FMS system provides broader, more uniform field distribution and deeper tissue penetration, potentially enhancing neuromuscular activation. These properties may account for the observed outcomes, as suggested by previous research in urinary incontinence and pelvic pain [16,17,29,30,31,32,33].

Importantly, the therapeutic effect appeared compartment-specific. Statistically significant gains were noted in the anterior compartment, whereas no meaningful changes were detected in the apical (C, D) or posterior (Ap, Bp) compartments. This may reflect either the anatomical specificity of the electromagnetic field distribution or the clinical profile of the study population, in which anterior compartment prolapse predominated. Additionally, the lack of improvement in deeper pelvic landmarks may indicate that modifications to session duration, frequency, or electromagnetic intensity could be necessary to achieve broader compartmental engagement. These observations warrant further investigation through imaging or electromyographic studies to confirm targeted muscular activation patterns during FMS.

These results align with a growing body of literature supporting the role of magnetic stimulation in pelvic floor dysfunctions such as stress and urge urinary incontinence [16,17,22,23,29,30,31,32,33]. Pelvic floor muscle training (PFMT) remains the gold standard for the conservative management of mild pelvic organ prolapse, supported by robust evidence from randomized trials and meta-analyses. A meta-analysis of 13 studies (*n* = 2340) demonstrated that women undergoing PFMT experienced significant improvements in prolapse symptoms (MD –3.07; 95% CI: –3.91 to –2.23) and POP stage severity (RR 1.70; 95% CI: 1.19–2.44) compared with controls [11]. Similarly, a randomized clinical trial showed that 45% of women in the PFMT group achieved an improvement in POP-Q stage versus 0% in the control group (*p* = 0.038), while 63% of participants reported subjective improvement compared with 24% in controls (*p* = 0.012) [8].

By contrast, the improvements observed with Flat magnetic stimulation (FMS) in the present study were of smaller magnitude and perceived as “minimal” by more than half of the patients. One potential advantage of FMS is that it offers a completely passive modality, which may enhance adherence among women unable or unwilling to perform regular exercises. Nevertheless, the absence of long-term data and the modest short-term anatomical changes observed in our cohort prevent direct equivalence with PFMT in terms of clinical efficacy. It is therefore plausible to consider FMS as a complementary rather than a substitutive option, particularly in women with poor compliance to PFMT or with physical limitations that hinder active training. Future prospective randomized controlled trials directly comparing FMS with supervised PFMT are warranted to establish whether magnetic stimulation can provide comparable or additive benefits to the established standard of care.

The present study has several important limitations. The absence of a sham or control group represents a major weakness, as it precludes definitive attribution of the observed improvements solely to the intervention. This limitation is largely inherent to the retrospective design, which was conceived with exploratory purposes in a real-world clinical setting, rather than within a randomized controlled framework. The short follow-up period of one week was intentionally selected to minimize dropouts, maximize adherence, and provide a complete dataset for safety and feasibility assessment. While this design ensured robust short-term data acquisition, it does not allow conclusions regarding the durability of treatment effects.

Furthermore, the clinical relevance of the small anatomical changes observed (e.g., Aa and Ba shifting from −0.3 to −0.7 cm) remains uncertain. While statistically significant, such millimetric differences may not necessarily translate into meaningful functional improvement for patients. These findings should therefore be interpreted with caution and considered as preliminary signals of efficacy rather than definitive therapeutic benefit. Moreover, more than half of the patients in our cohort described themselves as only “minimally improved.” This distribution of subjective responses highlights the modest nature of the clinical effect, despite the high overall proportion of women reporting some degree of improvement. It is possible that these outcomes reflect both the early stage of prolapse in our sample and the short follow-up window, where deeper or longer-lasting improvements could not be captured. Nonetheless, the predominance of minimal improvement tempers the overall strength of the findings and further reinforces the need for prospective, controlled trials to establish whether Flat Magnetic Stimulation can achieve clinically meaningful and durable benefits.

## 5. Conclusions

Flat magnetic stimulation, delivered through the Dr. Arnold system, demonstrates promise as a conservative, non-invasive treatment for women with mild pelvic organ prolapse. The modality yielded significant short-term anatomical improvements in the anterior vaginal wall, was well tolerated by all participants, and was associated with a high level of subjective satisfaction. Its ease of use, lack of discomfort, and favorable safety profile support its potential as an adjunct to or replacement for traditional conservative therapies such as PFMT. Nonetheless, robust prospective research is required to delineate long-term outcomes, identify optimal candidates, and position FMS within the broader therapeutic algorithm for pelvic floor disorders.

## Figures and Tables

**Table 1:** Baseline demographic and clinical characteristics of the study population (*n* = 87).

Variable	Value (Mean ± SD or *n*, %)
Number of participants	87
Age, years	59.1 ± 6.4 (range: 30–75)
Parity (number of vaginal births)	2
Body mass index (BMI), kg/m^2^	23.4 ± 2.6
POP stage (POP-Q)	All stage ≤ 2
Predominant compartment	Anterior vaginal wall

**Table 2:** Anatomical outcomes: POP-Q point measurements before and after Flat Magnetic Stimulation (FMS).

POP-Q Point	Baseline (Mean ± SD)	Post-Treatment (Mean ± SD)	*p*-Value
Aa	−0.3 ± 1.2	−0.7 ± 1.3	0.03
Ba	−0.3 ± 1.3	−0.7 ± 1.3	0.04
C	−5.4 ± 2.5	−5.5 ± 2.4	0.59
Ap	−1.1 ± 1.3	−1.2 ± 1.4	0.42
Bp	−1.2 ± 1.4	−1.3 ± 1.5	0.38

## Data Availability

The data presented in this study are available upon request from the corresponding author.
